# A deep‐learning framework for the prediction of the type of adaptive strategy of MR‐guided prostate radiotherapy

**DOI:** 10.1002/acm2.70395

**Published:** 2025-12-29

**Authors:** Wenlong Xia, Bin Liang, Kuo Men, Ke Zhang, Yuan Tian, Ningning Lu, Jianrong Dai

**Affiliations:** ^1^ National Cancer Center/National Clinical Research Center for Cancer/Cancer Hospital Chinese Academy of Medical Sciences and Peking Union Medical College Beijing China

**Keywords:** adaptive strategy, deep learning, deformable image registration, MR‐guided adaptive radiotherapy

## Abstract

**Background and purpose:**

In MR‐guided adaptive radiotherapy (MRgART), adaptive strategies are currently mainly determined through subjective review of anatomical changes. Machine learning (ML) models based on deformable image registration (DIR) have been developed to predict type of adaptive strategy: adapt to position (ATP) versus adapt to shape (ATS). However, subjective review may result in sub‐optimal plans and DIR processing for ML models can be time‐consuming. This study aims to develop a deep learning (DL) model that uses images as input data for fast and accurate adaptive strategy selection.

**Methods:**

Data from 180 fractions of 36 prostate cancer patients were used retrospectively for this study. The optimal adaptive strategy was determined between ATP and ATS according to dosimetric evaluation. A multi‐stage network method was proposed and used for adaptive strategy prediction. A DL‐based image registration (DLIR) network was first trained to register the reference image to the daily image. Then, a DL‐based adaptive strategy prediction (DLSP) model was constructed and trained using the encoder section of the trained DLIR network. Data from 24 patients were used for training, while the data from the remaining 12 patients formed the independent test set.

**Results:**

The DLSP model demonstrated high performance with an area under the curve (AUC) value of 0.861, and corresponding accuracy (ACC), sensitivity (SEN), and specificity (SPC) were 0.867, 0.898, and 0.727, respectively. The DL prediction process required approximately 2.5 min, representing a 5‐fold improvement in efficiency over the existing ML method.

**Conclusions:**

The DL‐based model could provide fast and accurate adaptive strategy selection, which further improves the efficiency of the MRgART process.

## 1 INTRODUCTION

Magnetic resonance (MR) imaging has several advantages such as excellent contrast for soft tissues, no exposure to extra radiation, and the ability to perform multi‐sequence functional imaging. The integration of MR imaging and linear accelerator (MR‐linac) was developed to realize MR‐guided adaptive radiotherapy (MRgART),^1^, ^2^, ^3^ and enable visualization and adjustment of anatomical changes during radiotherapy. In MRgART, adaptive plans can be generated online/offline according to inter/intra‐fractional changes in patient anatomy. Previous studies showed the feasibility of MRgART and the potential to reduce planning target volume (PTV) margins and achieve better normal tissue sparing.^4^, ^5^, ^6^, ^7^

In the workflow of MRgART, MR images are acquired as daily images while the patient is in the treatment position. These images are then used for soft tissue‐based position verification or online re‐planning. Commercial MR‐linacs offer different adaptive strategies, such as adapt to position (ATP) and adapt to shape (ATS) for the Elekta (Stockholm, Sweden) Unity system, optimization with segment weights and full re‐optimization for the ViewRay MRIdian (Oakwood Village, USA).^8^, ^9^ However, for the selection of different strategies, there is a trade‐off to be made between time spent adjusting the treatment plan against the dosimetric gain. Strategies to provide better quality treatment plans typically require a substantially longer treatment time and greater human effort.

The selection of the adaptive strategy is directly influenced by the deformation of anatomical structures between the daily image and the reference image. Currently, physicians and physicists determine the adaptive strategy in several ways. One approach is to visually review anatomical changes and make a decision in real‐time. However, visual inspection is limited by time, as well as by the level of expertise in anatomy, which can result in sub‐optimal plans during online treatment. Tyran et al. found that visual inspection of daily images was unreliable, and recommended using a prediction plan based on deformable image registration (DIR) to determine the adaptive strategy.^10^ Another approach is to build prediction models based on the anatomical changes. For instance, Lim et al. constructed a decision tree based on Jacobian determinant histogram (JDH) metrics obtained from DIR between the daily CT and reference CT.^11^ However, the improvement in prediction performance was limited because only simple statistical indicators of JDH metrics were used. In a previous study, we improved predictive performance using machine learning (ML) prediction models based on features derived from the deformation vector field (DVF).^12^ These features were obtained through a conventional (manual) DIR process and then filtered to create prediction models. However, the process of manually performing DIR and filtering features can be time‐consuming, leading to decreased clinical efficiency.

In this study, we propose a DL method directly using paired CT–MR images for rapid, accurate adaptive strategy selection. The DL prediction model was developed from a DL‐based VoxelMorph framework,^13^ which uses a convolutional neural network (CNN) to learn a parametrized registration function and optimizes the CNN parameters on a set of images.

## 2 METHODS

### 2.1 MRgART workflow using prediction model

**FIGURE 1:**
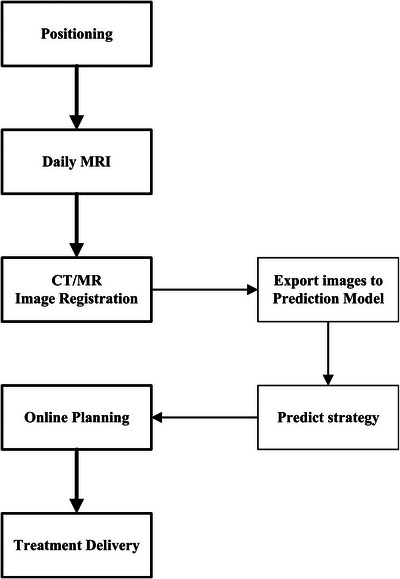
Clinical workflow of MRgART using adaptive strategy prediction model.

The clinical workflow of MRgART using a prediction model is shown in Figure 1. It includes several steps: positioning, daily MRI, CT–MR image registration, online planning, and treatment delivery. The procedure for adaptive strategy selection using prediction model based on the past treatment data was performed between the stages of image registration and online planning. Positioning was the initial step in the clinical workflow. Once the patient was positioned, a daily MR scan was performed. During the image registration process, the daily MR image and reference CT image were then exported to the prediction model. Based on the predicted adaptive strategy, an online treatment plan was created. Finally, the treatment plan was delivered.

### 2.2 Patient data and data preparation

This study enrolled 36 prostate cancer patients. All patients were treated with intensity‐modulated radiation therapy (IMRT) using 7‐MV photon beams at 425 MU/min on the 1.5T Unity MR‐linac system. The average total treatment time was 48 min, including 10 min of beam‐on time. For intra‐fractional motion monitoring, cine MR with simultaneous tri‐planar imaging (axial, coronal, and sagittal) was utilized.

The clinical target volume (CTV) was defined as the whole prostate, while the planning target volume (PTV) was defined as the CTV plus a 3 mm isotropic margin. A simultaneous boost of CTV4000 was defined as the prostate with a 1 mm contraction for the high‐risk region. All critical organs at risk (OARs), including the bladder, rectum, urethra, and bilateral femoral heads, were delineated. The prescription doses for PTV and CTV4000 were 36.25 Gy and 40.00 Gy, respectively, in five fractions treated every other day.

The patients underwent CT scan with no contrast in a supine position with 3‐mm slice thickness and 1 mm × 1 mm voxel size. Daily MR images (T2‐weighted) were acquired with 1‐mm slice spacing. Pretreatment plans and adaptive plans were generated using the Monaco (V5.40, Elekta AB, Stockholm, Sweden) treatment planning system. Both ATP plans and ATS plans using 7–10 IMRT fields were generated for 180 fractions. All 360 plans were designed according to the dose–volume criteria in PACE‐B trial^14^ and normal tissue dose constraints of stereotactic radiotherapy.^15^ All treatment fractions were delivered using ATS plans. The ATP plan of each fraction was re‐calculated in offline mode for the same MR image used for the ATS plan. As in previous study,^12^ the ground‐truth strategies were determined by physicians based on the evaluation results of the re‐calculated ATP plans and ATS plans according to the criteria listed in Table 1. For all fractions, the reference CT and daily MR were used as the reference image and daily image, respectively. A region of interest (ROI) mask was generated for each fraction by uniformly expanding the PTV by 50 mm. This mask was applied during model training to focus the analysis on the most relevant anatomical area and mitigate the influence of registration errors in distant regions.

**TABLE 1:** Dosimetric criterion and tolerance for MRgART of prostate cancer.

Structure	Dosimetric criterion	Tolerance
**CTV4000**	V_40Gy_ > 95%	−5%
**PTV**	V_36.25 Gy_ > 95%	−5%
	V_34.40 Gy_ > 98%	−5%
**Rectum**	D_max_ < 40 Gy	0 Gy
	V_38Gy_ < 0.1cc	0cc
	V_36Gy_ < 1cc	+1cc
	V_29Gy_ < 20%	+1%
**Bladder**	V_37Gy_ < 10cc	+0.5cc
	V_18.1 Gy_ < 50%	+2.5%
**Femur R**	V_14.5 Gy_ < 5%	+0.5%
**Femur L**	V_14.5 Gy_ < 5%	+0.5%
**Urethra**	D_50%_ < 41 Gy	+1.5 Gy

### 2.3 DL‐based image registration (DLIR) model

**FIGURE 2:**
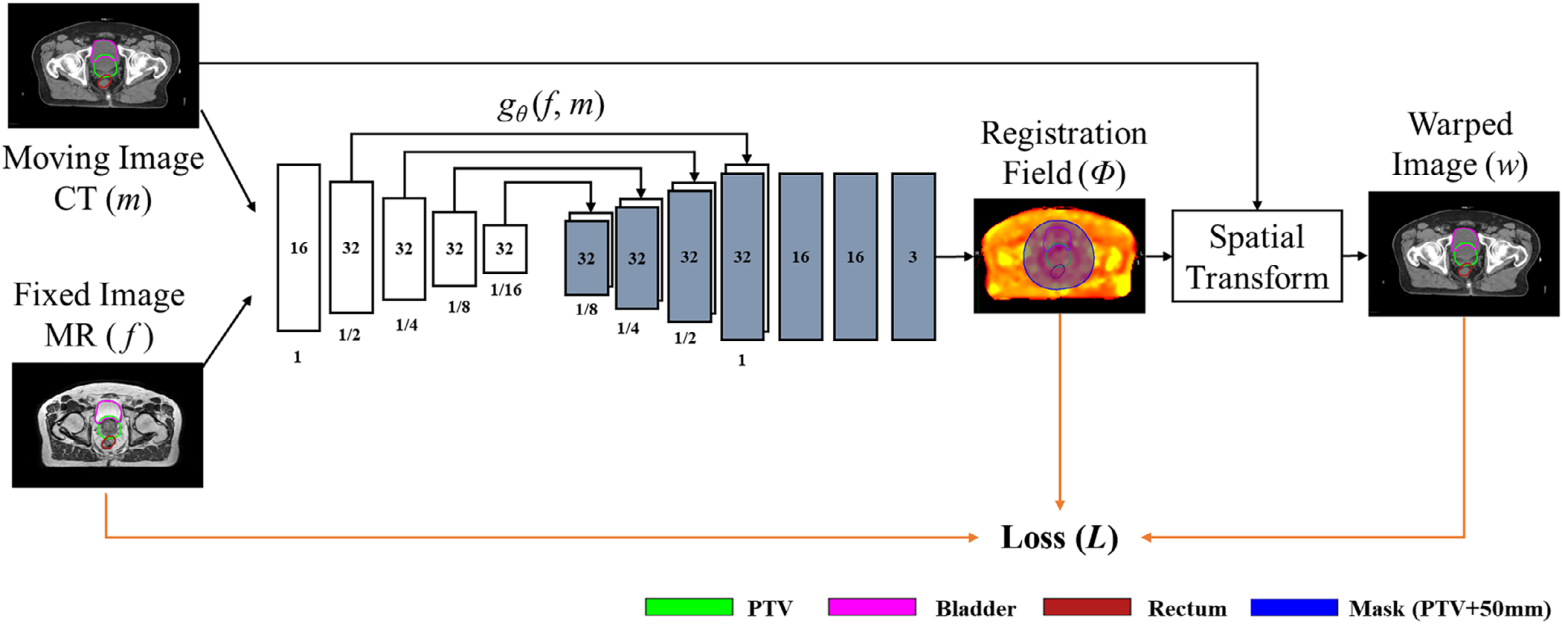
Overview of the DLIR model.

We proposed a multi‐stage network method for the adaptive strategy prediction. In the first stage, a DLIR model was developed based on the VoxelMorph framework.^13^ The network requires the paired input images in the same dimension and aligned properly. Therefore, the original paired CT–MR images were pre‐processed for resampling and affine transformation (rigid registration) using a quick pre‐defined workflow in MIM software (Cleveland, OH, USA). Figure 2 presents an overview of the DLIR network.

The network takes a fixed image (*f*, the daily image) and a moving image (*m*, the reference image) as input. A convolutional neural network (CNN) defines a function *g_θ_* (*f*, *m*), where *θ* represents the kernels of the convolutional layers. This function computes a registration field *Φ*, which maps the coordinates of the fixed image to those of the moving image. The moving image is warped using *Φ* and a spatial transformation function to generate a warped image (*w*). The similarity between *w* and *f* is then evaluated.

The *g_θ_* function is parameterized using a UNet CNN architecture, which includes both encoder and decoder sections connected by skip connections.^16^, ^17^ A single input is used by concatenating *m* and *f* into a 2‐channel 3D image with a size of 256 × 256 × 128 × 2. Both the encoder and decoder sections apply 3D convolutions with a kernel size of 3 and a stride of 2. The convolutional layers extract hierarchical features from the input image pairs to estimate *Φ*. In the encoder section, strided convolutions reduce the spatial dimensions by half at each layer. In the decoder section, up‐sampling, convolutions, and skip connections are alternated to propagate the features learned in the encoder section directly to the layer that generates the registration. The UNet configuration employs 4‐layer encoder and decoder sections, with skip connections implemented through concatenation of encoder features to corresponding decoder levels. All intermediate convolutional layers use LeakyReLU (*α *= 0.2) activations, while the final displacement field output uses linear activation.

As shown in Equations (1)–(4), we used the same loss function (*L_DLIR_)* as that proposed by Balakrishnan.^13^ *L_DLIR_* consists of three components:

(1) $$L_{\mathrm{DLIR}}=L_{\mathrm{sim}}+\lambda_{1}L_{\mathrm{smooth}}+\lambda_{2}L_{2}$$

(2) $$L_{sim}=-\sum_{y\in f}\sum_{x\in w}P_{\left(f,w\right)}\left(x,y\right)\log\left(\frac{P_{\left(f,w\right)}\left(x,y\right)}{P_{f}\left(x\right)P_{w}\left(y\right)}\right)$$

(3) $$L_{smooth}=\sum_{u\in \Phi }{\left\|\nabla u\right\|}^{2}=\sum_{u\in \Phi }{\left\|\frac{\partial u}{\partial x},\frac{\partial u}{\partial y},\frac{\partial u}{\partial z}\right\|}^{2}$$

(4) $$L_{2}=\sum_{u\in \Phi }{\left\|u\right\|}^{2}$$

*L_sim_* is the negative value of mutual information (MI) of the fixed (*f*) and warped (*w*) image. *P*_(_ *_f,w_*_)_(*x,y*) is the joint probability mass function of *f* and *w. P_f_* (*x*) and *P_w_* (*y*) are the marginal probability mass functions of *f* and *w*, respectively. MI measures the similarity of images: the greater, the more similar. Negative MI was used to penalize differences in appearance. Another consideration of using negative MI was to keep it consistent with the remaining two terms of *L_DLIR_*. So *L_DLIR_* could be minimized for training. *L_smooth_* penalizes the non‐smooth *Φ*, which is not physically realistic. It is calculated as the 2‐norm of the spatial gradients of displacement (*u*). *L_2_* is the 2‐norm of *u*, that prevents overfitting by favoring smaller parameters over larger ones. *λ_1_* and *λ_2_* are the weights, which were set to 10^−2^ and 10^−5^, respectively. The DLIR model was trained using the Adam optimizer with learning rate of 1e^−4^ for 200 epochs. The batch size was set to 1 due to memory constraints.

During the training process of the DLIR model, the mask region (PTV+50 mm) was not used. The loss function (*L_DLIR_*) was calculated for the entire CT‐MR images and used to assess the model's performance.

### 2.4 DL‐based adaptive strategy prediction (DLSP) model

**FIGURE 3:**
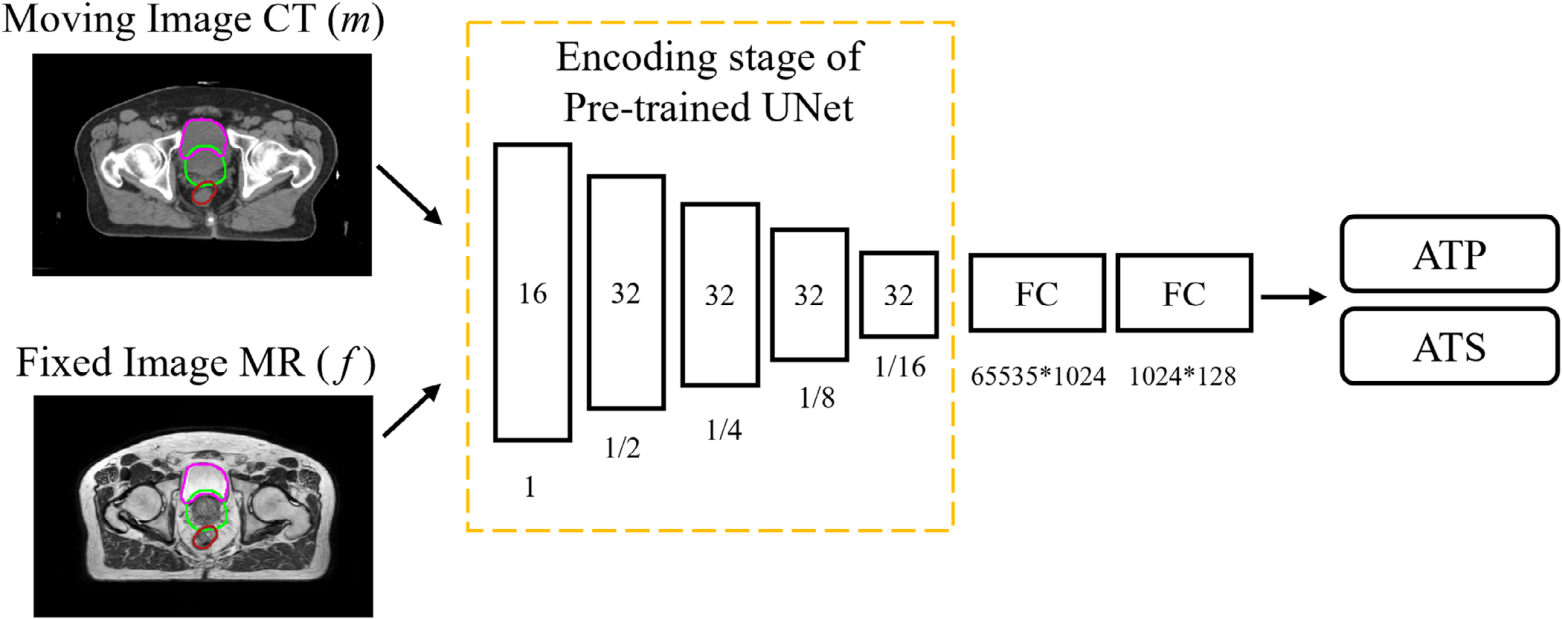
Overview of the DLSP model.

In the second stage, we developed a DLSP model based on the trained DLIR model. The architecture is shown in Figure 3. The DLSP model utilizes encoder features from the DLIR network to capture deformation patterns. These features encode spatial relationships critical for strategy prediction.

The DLSP model consists of two modules: the encoder section of DLIR and two cascaded fully connected (FC) layers. The mask region (PTV+50 mm) was utilized to mitigate any unnecessary influence from external regions. The DLSP model used the same input as DLIR model. The weights of the encoder section were transferred from the pre‐trained DLIR model and frozen during the training of the DLSP model. The binary cross entropy (BCE) loss function was used (Equation 5):

(5)

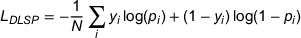

where *y_i_* is the label, 1 for ATS and 0 for ATP. *p* is the probability generated by the DLSP model. The DLSP model was trained using the Adam optimizer with learning rate of 1e^−5^ for 300 epochs and a batch size of 2.

### 2.5 Model evaluation

Data from 24 patients treated prior to March 2022 were used for training, while data from 12 patients treated after March 2022 formed the independent test set. Patient‐level data splitting prevented inter‐fraction dependency bias and cross‐contamination across datasets. The prediction model was evaluated using the indices of area under the curve (AUC), accuracy, sensitivity, specificity, and F1 score. F1 score is an error metric defined as 2TP/(2TP+FP+FN), where TP is true positive, FP is false positive, and FN is false negative. The processing time of the DL‐based adaptive strategy prediction model was counted and compared to the existing ML model.^12^ The training and testing for all models were conducted on a system with the following hardware configuration: an NVIDIA GeForce RTX 4090 GPU with 24 GB of VRAM, an Intel Core i9‐13900K 3.0 GHz processor, and 64 GB of DDR5 RAM.

## 3 RESULTS

### 3.1 Evaluation results of patient data

Table 2 shows the results of adaptive strategy selection for 180 fractions from 36 patients. ATP was selected in 23% and ATS in 77% of the fractions, based on physician evaluation. These were used as the ground truth for model training. The ground‐truth adaptive strategies for each treatment fraction were determined through dosimetric evaluation, as detailed in Section 2.2. This indicates potential to improve the efficiency of MRgART for prostate cancer.

**TABLE 2:** Adaptive strategy selection results for 180 fractions of 36 patients.

Number of Patients	Number of fractions ATP/ATS
17	0/5
9	1/4
4	2/3
2	3/2
1	4/1
3	5/0
**Total**	**42/138**

### 3.2 Results of pretrained DLIR model

**FIGURE 4:**
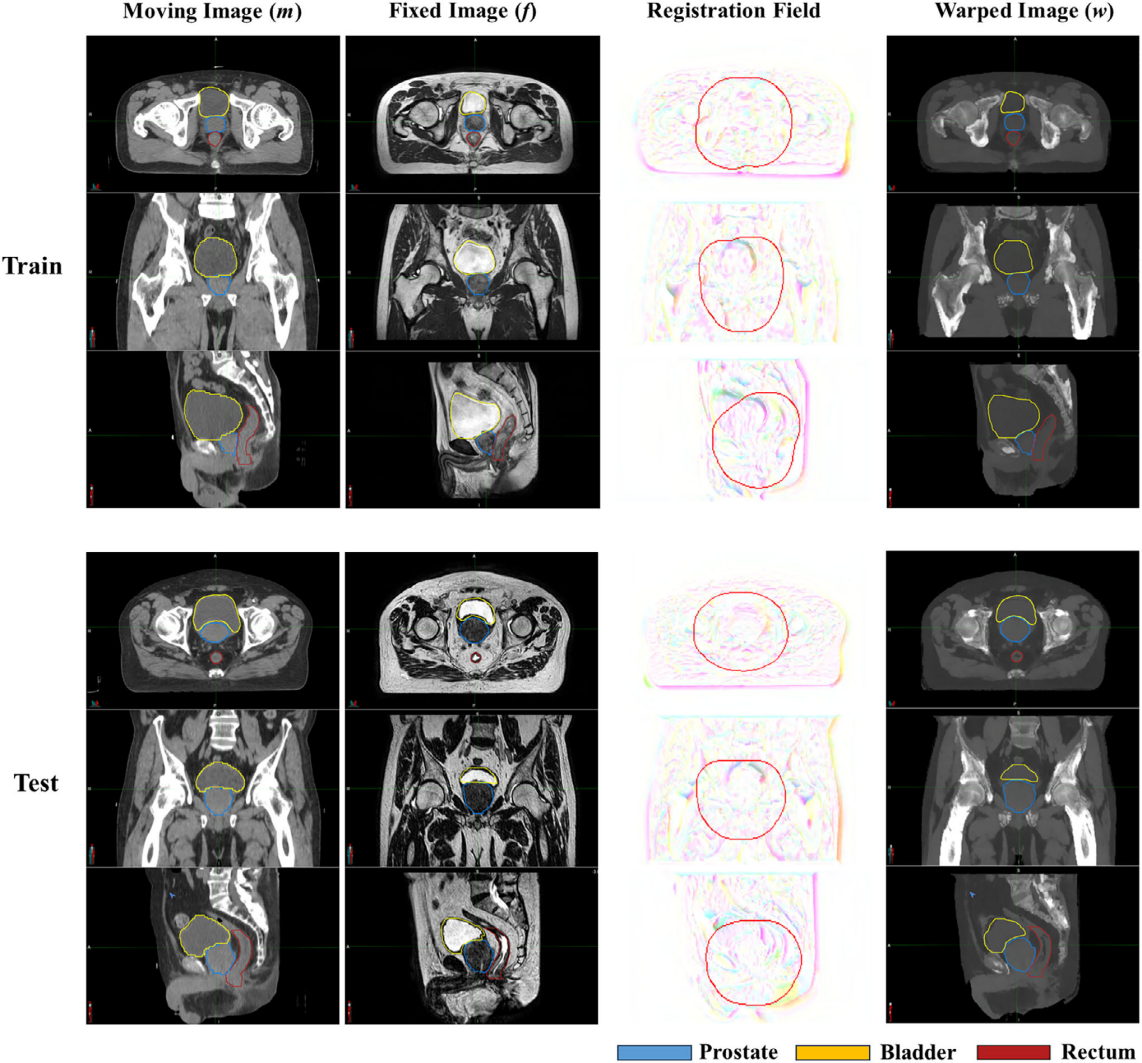
Example visual results of the warped images from training set (above) and testing set (below) using the pretrained DLIR network, the mask regions are depicted as red contours on the registration fields.

**FIGURE 5:**
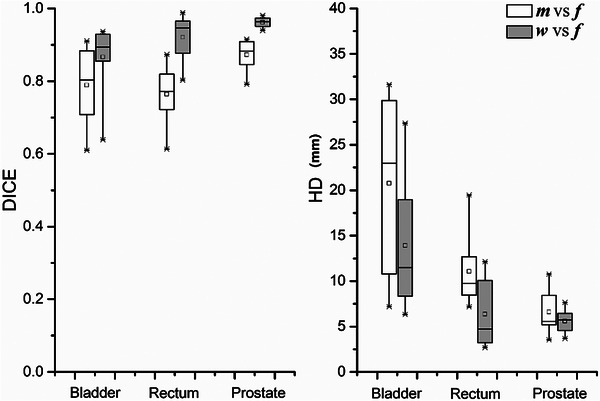
Quantitative evaluation of the DLIR model's performance. Dice coefficient and Hausdorff distance before prediction: moving (*m*) vs fixed (*f*) images and after prediction: warped (*w*) vs. fixed (*f*) images using trained DLIR model. The significant improvement in DSC and reduction in HD after registration quantitatively validates the model's ability to accurately align the daily and reference images. The box plots display the first and third quartiles (boxes), medians (bands inside), average values (small boxes inside), standard deviations (whiskers), and outliers (crosses).

**FIGURE 6:**
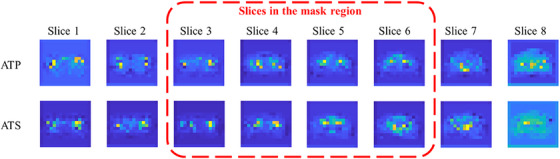
Feature maps from the UNet encoder's final layer. The variation in these 16×16×8 activations reflect the diverse features extracted from the input, which encode anatomical deformations for the subsequent DLSP prediction task.

Figures 4, 5, 6 collectively validate the DLIR model's performance. Figure 4 presents warped image results demonstrating successful CT‐MR registration. Figure 5 quantifies anatomical alignment improvements through Dice coefficients and Hausdorff distances calculated preregistration (moving vs. fixed images) and postregistration (warped vs. fixed images). Figure 6 visualizes the 16 × 16 × 8 feature maps from the UNet encoder's final layer, confirming effective feature extraction for downstream prediction. These results provide comprehensive evidence of the model's registration accuracy, quantitative performance gains, and feature learning capability.

### 3.3 Evaluation results of DLSP model

**FIGURE 7:**
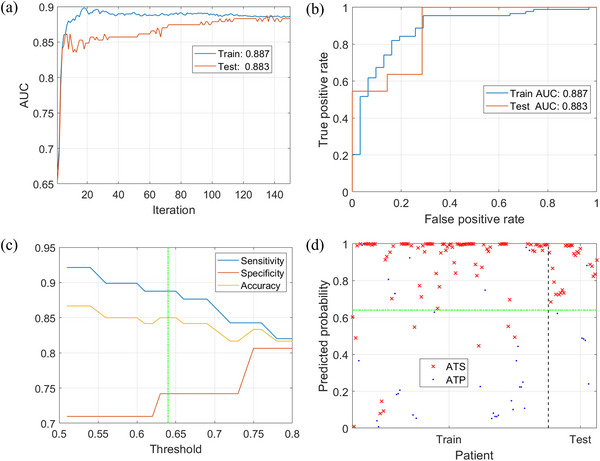
Training and evaluation results of the DLSP model. (a) The AUC convergence curve shows the model's learning progression and stability on the test set after ∼100 iterations. (b) The receiver operating characteristic (ROC) curve for the test set, with an AUC of 0.861, demonstrates the model's high discriminatory power in classifying adaptive strategies. (c) The sensitivity–specificity trade‐off across different prediction thresholds guides the selection of an optimal operating point. (d) The predicted probability distributions for the training and test sets, with the decision threshold set at 0.64, visually show the model's clear separation between ATP and ATS cases, confirming its robust predictive performance.

Figure 7a shows the change of AUC value with the number of iterations during the training process of the DLSP model. The test set tends to stabilize and converge from 100 iterations. Figure 7b shows the receiver operating characteristic (ROC) curves of the DLSP model. The results showed good performance with a test AUC value of 0.861. Figure 7c presents the sensitivity and specificity of the DLSP model vary with threshold. The DLSP model outputs a probability for each case. A threshold of 0.64 was selected to balance accuracy, specificity, and sensitivity. Figure 7d shows the predicted probability distributions for both training and test sets, with the threshold set at 0.64. The DLSP model computes a probability for each case. If the probability is greater than the threshold, ATS will be chosen. On the other hand, if the probability is smaller than the threshold, ATP will be chosen.

Table 3 lists the results for the indices used to evaluate the prediction performance of the DLSP model: AUC, accuracy, specificity, sensitivity, and F1 score.

**TABLE 3:** Results of the prediction performance of the DLSP model.

Method	Dataset	AUC	Accuracy	Sensitivity	Specificity	F1 score
**DLSP model**	**Training**	0.887	0.850	0.888	0.742	0.898
	**Test**	0.861	0.867	0.898	0.727	0.804

**FIGURE 8:**
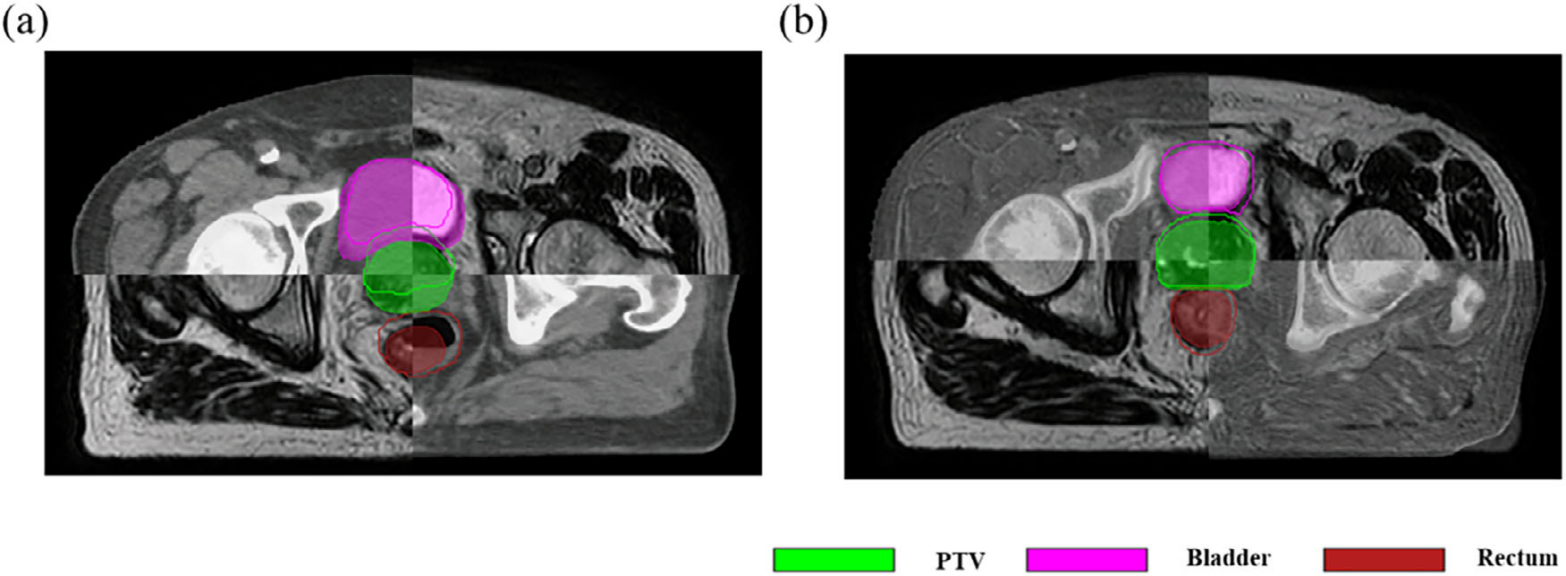
Image registrations for fractions predicted to use the ATS adaptive strategy: (a) Successful case (high deformation); (b) Failure due to registration errors from low soft‐tissue contrast. Contours derived from the reference CT and daily MR are shown as poly lines and color wash, respectively.

Figure 8 visualizes image registration outcomes for ATS‐predicted fractions. For treatment fractions where predictions succeeded (Figure 8a), image comparisons revealed more pronounced deformation magnitudes in the prostate and rectal regions. Conversely, prediction failures (Figure 8b) occurred in fractions where deformations were less significant, primarily due to multi‐modal registration errors caused by insufficient soft‐tissue resolution or large anatomical deformations. These misalignments directly caused model failure. A potential solution to enhance prediction accuracy is to convert multi‐modal images into a unified modality using a convolutional neural network (CNN) before registration.

**FIGURE 9:**
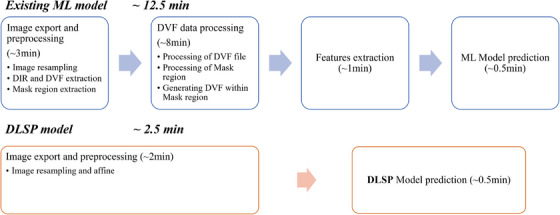
Clinical workflow for the existing ML model and the DLSP model.

Figure 9 shows the clinical workflows of the DLSP model and the existing ML model. Processing DVF data and extracting features in the existing ML model can be time‐consuming, potentially reducing the efficiency of clinical treatment. In contrast, the DLSP model uses a quick pre‐defined workflow to pre‐process the original image pairs for image resampling and affine transformation. The processing time of the DLSP model and the existing ML model^12^ were about 2.5 min and 12.5 min, respectively.

## 4 DISCUSSION

Due to organ movement in MRgART treatment, inter‐fraction organ deformation and displacement can vary unpredictably.^18^, ^19^ The selection of an adaptive strategy is closely related to the DVF between the reference image and daily image. As mentioned before, Lim et al. used a decision tree based on JDH metrics to quickly determine the need for online replanning, achieving an AUC of 0.76.^11^ We built ML prediction models using DVF features to select adaptive strategies, achieving superior performance with an AUC of 0.896.^12^ However, the ML prediction models mentioned in above studies required data preprocessing such as manual DIR and feature filtering. This can be a time‐consuming process and may result in decreased clinical efficiency. In this study, we developed a novel DL framework that achieved a high predictive performance (AUC = 0.861). The daily and reference images can be directly input into the DLSP model. Compared to our previous ML model,^12^ the DLSP model significantly reduced the processing time of the clinical workflow from 12.5 to 2.5 minutes, increasing clinical efficiency by five times.

The clinical workflow of MRgART involves several steps, including positioning, daily MR imaging, image registration, online planning, and treatment delivery. Adaptive strategy selection using model prediction could be performed between the image registration and online planning stages. Implementation of the adaptive strategy prediction model may reduce unnecessary adoption of ATS strategies, thereby shortening contour adjustment and online replanning durations. Compared to previous ML models, our results suggest that the DLSP model could effectively assist physicians in quickly and accurately determining when the ATS adaptive strategy is beneficial for specific fractions and when it is unnecessary. Therefore, the use of the DLSP model in the clinic could significantly improve the efficiency of the MRgART process.

Although the DLSP model was trained using prostate cancer patients, the methodological framework is generalizable. Applying this approach to other tumor sites or imaging modalities would require training a new model on a relevant dataset but would not necessitate fundamental architectural changes. ​​Furthermore, the method proposed in this study is not limited to MRgART and has broader applications in adaptive radiotherapy (ART), such as CBCT or CT‐guided adaptive radiotherapy. The ATP and ATS strategies are equivalent to repositioning and replanning in conventional linacs, respectively. For systems that do not use the ATP/ATS approach, this method can be easily adapted to determine the need for online replanning based on the guide and reference images.

In this study, the DLIR and DLSP models were trained and tested using 36 patients, which was 16 more than the ML model in the previous study. To avoid bias in the patient data for the two methods, we re‐trained and re‐tested the multi‐stage network using the same 20 patients as in the ML method. The results are presented in the supplementary material, Figure S1 and Table S1. Figure S1 presents: (a) the stable AUC convergence after ∼20 training iterations; (b) ROC curves confirming test AUC (0.847); (c) sensitivity–specificity tradeoffs across prediction thresholds; and (d) probability distributions for ATP/ATS strategy selection using a 0.64 decision threshold. Table S1 details performance metrics, revealing consistent training/test results. According to the results, the DLSP model using 20 patients achieved AUC value of 0.847, and the performance was inferior to the ML model and the DLSP model in this study. Generally, DL models often require large amounts of data to train effectively. This is because they have many more parameters than ML models and need more data to accurately learn the relationships between the input and output.^20^ The increased patient data improved the performance of the multi‐stage network for adaptive strategy prediction.

Since the DVF from DIR process between the reference image and daily image was the main factor in determining an adaptive strategy, we selected the VoxelMorph framework for image registration as the first stage of our multi‐stage network method. The DLIR network was developed from the VoxelMorph framework, which was originally designed to accelerate medical image analysis using same‐modality 3D MR brain scans.^13^ Since our goal was to register multi‐modality images involving CT and MR, the registration task in this study was more complex. As demonstrated in Figures 4 and 5, the pre‐training results of the registration network show good performance for the prostate and rectum. The registration performance for the bladder was inferior to that for the prostate and rectum, especially in cases of large deformation. This is likely due to the inherent challenges of multi‐modal (CT–MR) registration and lower soft‐tissue contrast in the bladder region on these imaging sequences. Future work could investigate the use of generative networks to synthesize one modality from the other to create a mono‐modal registration task. However, this study mainly focused on the encoder section of the UNet, which was extracted and used to construct the DLSP model. As shown in Figure 6, the output data from the encoder section of the pre‐trained UNet could provide sufficient features for the DLSP model. The model's performance, achieving an AUC of 0.861, also verified the feasibility of using the multi‐stage network approach. Deep learning for image registration (e.g., VoxelMorph, TransMorph) has been extensively explored and widely used in medical imaging. Adaptive strategy prediction model was trained based on the image registration model. The larger global receptive fields of the transformer‐based methods (e.g., TransMorph, ViT) could be adopted to improve registration for extreme anatomical changes, thereby enhancing performance in adaptive strategy prediction model.

Our study had several limitations. First, although we increased the patient data for the DL method, the sample size is still limited. This may reduce the model's generalization capability to unseen data, as limited data typically increases sensitivity to dataset‐specific variations. Collecting a larger dataset is prioritized to enhance the model's generalization performance and facilitate its future clinical deployment. Additionally, physiologically plausible data augmentation techniques can be employed to further improve robustness. Second, the number of fractions used to test the models were limited. Increasing the samples in the test set could further improve the specificity. Low specificity of the model reduces its ability to identify fractions appropriate for ATP strategies. This limitation may lead to unnecessary selection of ATS strategies for certain treatment fractions, prolonging treatment delivery time. However, ATS strategies intrinsically offer superior plan quality compared to ATP. When misclassification occurs, we prioritize retaining the plan quality advantages of ATS, despite the potential reduction in clinical workflow efficiency. Third, the DLIR network performed well for small‐scale deformation. However, for large‐scale deformation, the model did not meet our expectations. Although the encoder section provides efficient features for the DLSP model, improved performance of the DLIR network could further enhance the DLSP model's performance, making it more reliable for clinical use. To enhance DLIR model performance, we propose: (1) improved image pre‐processing through soft‐tissue contrast enhancement in original images, and (2) targeted data augmentation for extreme cases via synthetically generated deformation fields focused on bladder anatomy. At last, the DLSP network utilizes features from the encoder section of the U‐NET. However, it currently does not take into account factors such as margins and constraints that may affect adaptive strategy selection. A potential solution could be to incorporate additional dose distribution and the contours of critical organs into the model.

## 5 CONCLUSIONS

In summary, we have developed a new method for predicting the best choice of adaptive strategy (adapt to shape, ATS vs. adapt to position, ATP) in MRgART of prostate patients using DL‐based prediction models. Clinically applying this method could accurately predict the adaptive strategy and enable fast, automatic adaptive strategy selection, thereby improving the efficiency of the MRgART process.

## AUTHOR CONTRIBUTIONS

Wenlong Xia. and Bin Liang conceived the study, developed the deep learning framework, performed the analyses, and drafted the manuscript. Kuo Men and Ke Zhang contributed to the methodology, data processing, and model validation. Yuan Tian assisted with formal analysis and interpretation of results. Ningning Lu was responsible for clinical data collection. Jianrong Dai provided overall supervision, acquired funding, and critically revised the manuscript. All authors reviewed and approved the final version of the manuscript.

## CONFLICT OF INTEREST STATEMENT

The authors declare no conflicts of interest.

## ETHICAL APPROVAL AND CONSENT TO PARTICIPATE

This retrospective study was conducted in accordance with the Declaration of Helsinki and approved by the independent Ethics Committee of Cancer Hospital, Chinese Academy of Medical Sciences. The requirement for informed consent was waived by the ethics committee due to the anonymized nature of the data and retrospective study design, in compliance with national regulations and institutional policies.

## Supporting information



Supporting Information
