# Supplementary material for: A deep‐learning framework for the prediction of the type of adaptive strategy of MR‐guided prostate radiotherapy
Source: J Appl Clin Med Phys. 2025 Dec 29;27(1):e70395. doi: 10.1002/acm2.70395 (PMC12746477; doi:10.1002/acm2.70395)
Supplement: Supplementary file 1 — Supporting Information [file ACM2-27-e70395-s001.docx]

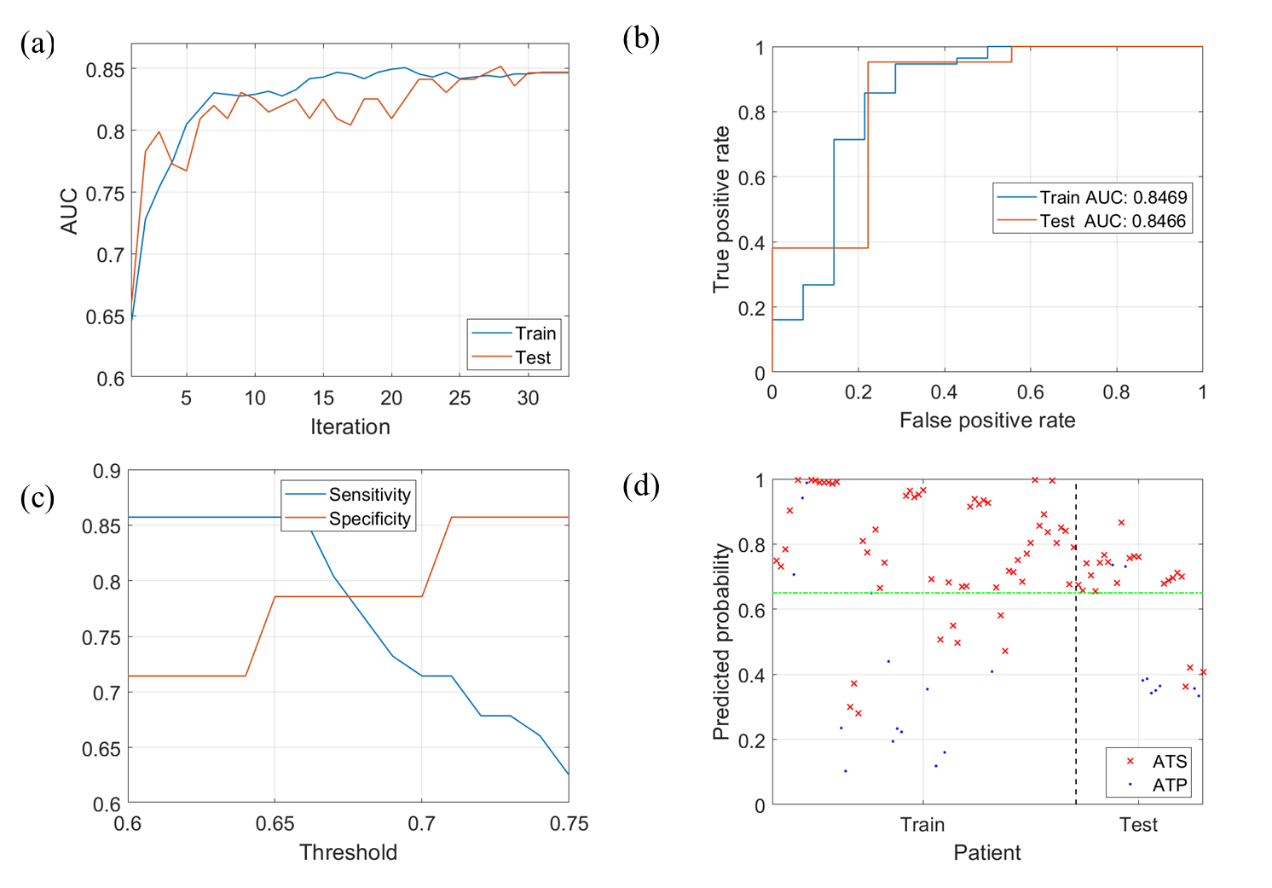


**Fig. S1.** (a) the change of AUC value with the number of iterations during the training process of the DLSP model; (b) the receiver operating characteristic (ROC) curves of the DLSP model; (c) the sensitivity and specificity of the DLSP model vary with threshold; (d) the predicted probability distributions for both training and test sets, with the threshold set at 0.64 (These results derive from the data presented in the previous study [12])

**Table S1.** Results of the prediction performance of the DLSP model (These results derive from the data presented in the previous study [12])

| **Method** | **Dataset** | **AUC** | **Accuracy** | **Sensitivity** | **Specificity** | **F1 score** |
| --- | --- | --- | --- | --- | --- | --- |
| **DLSP model** | **Training** | 0.847 | 0.843 | 0.857 | 0.786 | 0.897 |
|  | **Test** | 0.847 | 0.833 | 0.857 | 0.778 | 0.878 |
